# Supplementary material for: Chromosomal and gonadal sex have differing effects on social motivation in mice
Source: Biol Sex Differ. 2025 Feb 19;16:13. doi: 10.1186/s13293-025-00690-y (PMC11837725; doi:10.1186/s13293-025-00690-y)
Supplement: Supplementary file 1 — Supplementary Material 1. Figure 1: Open field assay revealed chromosomal and MYT1L genotype effects on activity. A) MYT1L genotype of all mice across 19 Myt1l+/- XX x Myt1l+/+ XYM litters, including experimental mice used in open field and social operant assays. MYT1L mutation was inherited by significantly fewer offspring than expected by mendelian heritability patterns. B) Sex factor breakdown of all mice across 24 litters, including experimental mice used in open field and social operant assays. The 24 litters included 19 Myt1l Het XX x Myt1l WT XYM litters, and 5 Myt1l WT XX x Myt1l WT XYM litters. Inheritance of modified third chromosome with Sry gene followed expected mendelian heritability. C) Diagram of open field chamber. Dashed red line designates boundary between center zone (orange) and perimeter zone (no color). D) XX mice travel a greater distance in the perimeter than XY mice. E) MYT1L Het mice travel a greater distance in the perimeter than MYT1L WT mice. F) XX mice travel a greater distance in the center than XY mice. G) MYT1L Het mice travel a greater distance in the center than MYT1L WT mice. H) MYT1L Het mice entered the perimeter significantly more than MYT1L WT mice. I) MYT1L Het mice entered the center significantly more than MYT1L WT mice. J) XX mice spend more time in the perimeter zone than XY mice. K) XX mice spend less time in the center zone than XY mice. L) XY mice spend more time on average per visit in the center zone compared to XX mice. In the full univariate model, sex chromosomes and gonads interact to influence mean time per visit in the center (p=0.048). Specifically, XX mice with testes typically spent less time per visit in the center while XY mice with testes spent significantly more time per visit in the center. M) MYT1L Het mice spend less time in the center per visit compared to MYT1L WT mice. For all panels, error bars indicate SEM. Asterisk (*) indicates variables that underwent square root transformation to normalize d [file 13293_2025_690_MOESM1_ESM.docx]

**Supplementary Figures:**

**
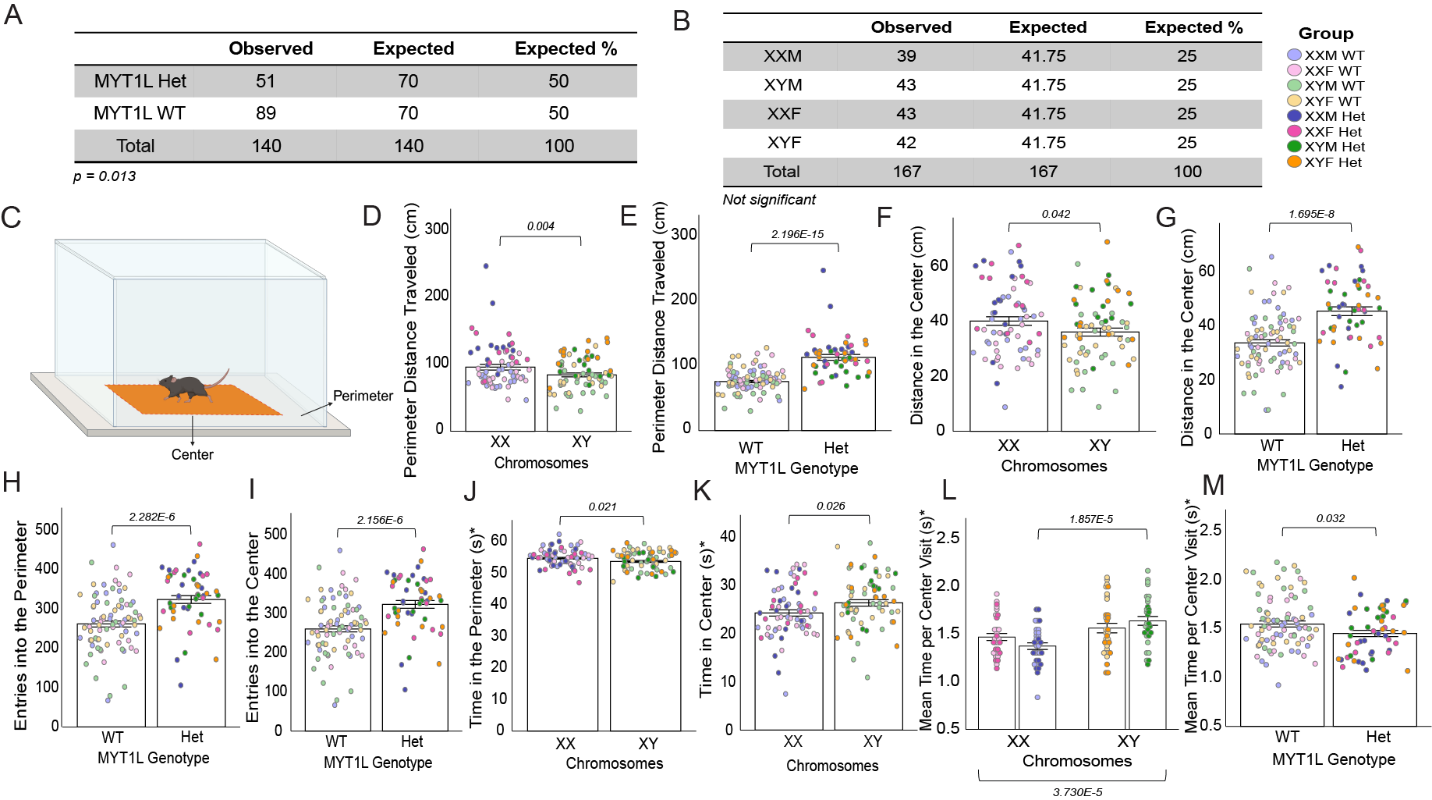
**

**Supplementary Figure 1: Open field assay revealed chromosomal and MYT1L genotype effects on activity. A)** MYT1L genotype of all mice across 19 *Myt1l^+/-^ XX x Myt1l^+/+^ XYM* litters, including experimental mice used in open field and social operant assays. MYT1L mutation was inherited by significantly fewer offspring than expected by mendelian heritability patterns. **B)** Sex factor breakdown of all mice across 24 litters, including experimental mice used in open field and social operant assays. The 24 litters included 19 *Myt1l* Het *XX x Myt1l* WT *XYM* litters, and 5 *Myt1l* WT *XX x Myt1l* WT *XYM* litters. Inheritance of modified third chromosome with *Sry* gene followed expected mendelian heritability. **C)** Diagram of open field chamber. Dashed red line designates boundary between center zone (orange) and perimeter zone (no color). **D)** XX mice travel a greater distance in the perimeter than XY mice. **E)** MYT1L Het mice travel a greater distance in the perimeter than MYT1L WT mice. **F)** XX mice travel a greater distance in the center than XY mice. **G)** MYT1L Het mice travel a greater distance in the center than MYT1L WT mice. **H)** MYT1L Het mice entered the perimeter significantly more than MYT1L WT mice. **I)** MYT1L Het mice entered the center significantly more than MYT1L WT mice. **J)** XX mice spend more time in the perimeter zone than XY mice. **K)** XX mice spend less time in the center zone than XY mice. **L)** XY mice spend more time on average per visit in the center zone compared to XX mice. In the full univariate model, sex chromosomes and gonads interact to influence mean time per visit in the center (*p=0.048)*. Specifically, XX mice with testes typically spent less time per visit in the center while XY mice with testes spent significantly more time per visit in the center. **M)** MYT1L Het mice spend less time in the center per visit compared to MYT1L WT mice. For all panels, error bars indicate SEM. Asterisk (*) indicates variables that underwent square root transformation to normalize data distribution.

**
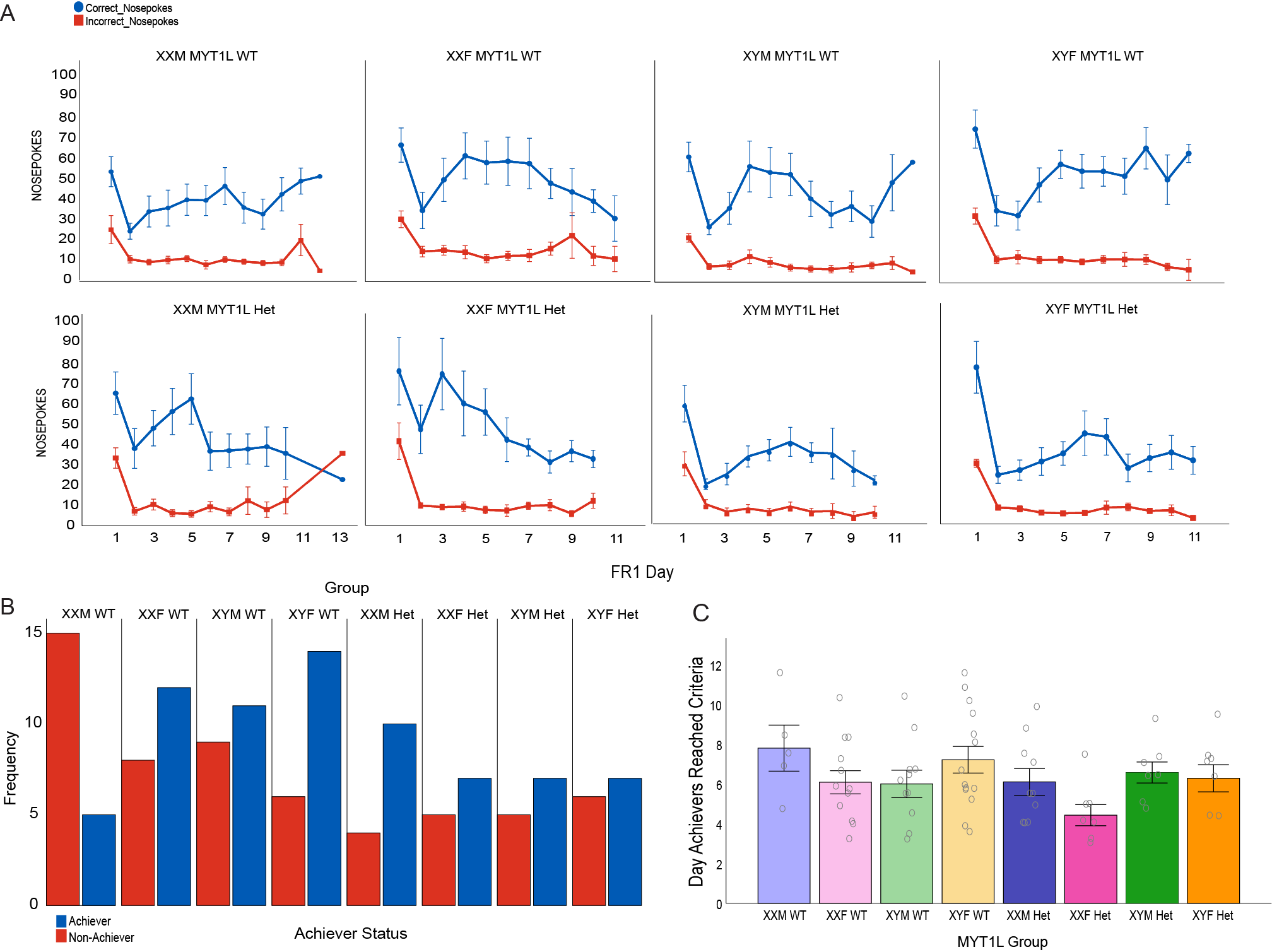
**

**Supplementary Figure 2: Conditioning achievement did not depend on MYT1L genotype or sex factors. A)** Mean of daily nosepokes across the FR1 testing period, with correct nosepokes in blue and incorrect nosepokes in red. Top row are MYT1L WT and bottom row are MYT1L Het **B)** Histogram showing frequency of achievers (blue) and non-achievers (red) per group. **C)** FR1 day achievers reached the third consecutive day of criteria across the eight experimental groups. For all panels, error bars indicate SEM.


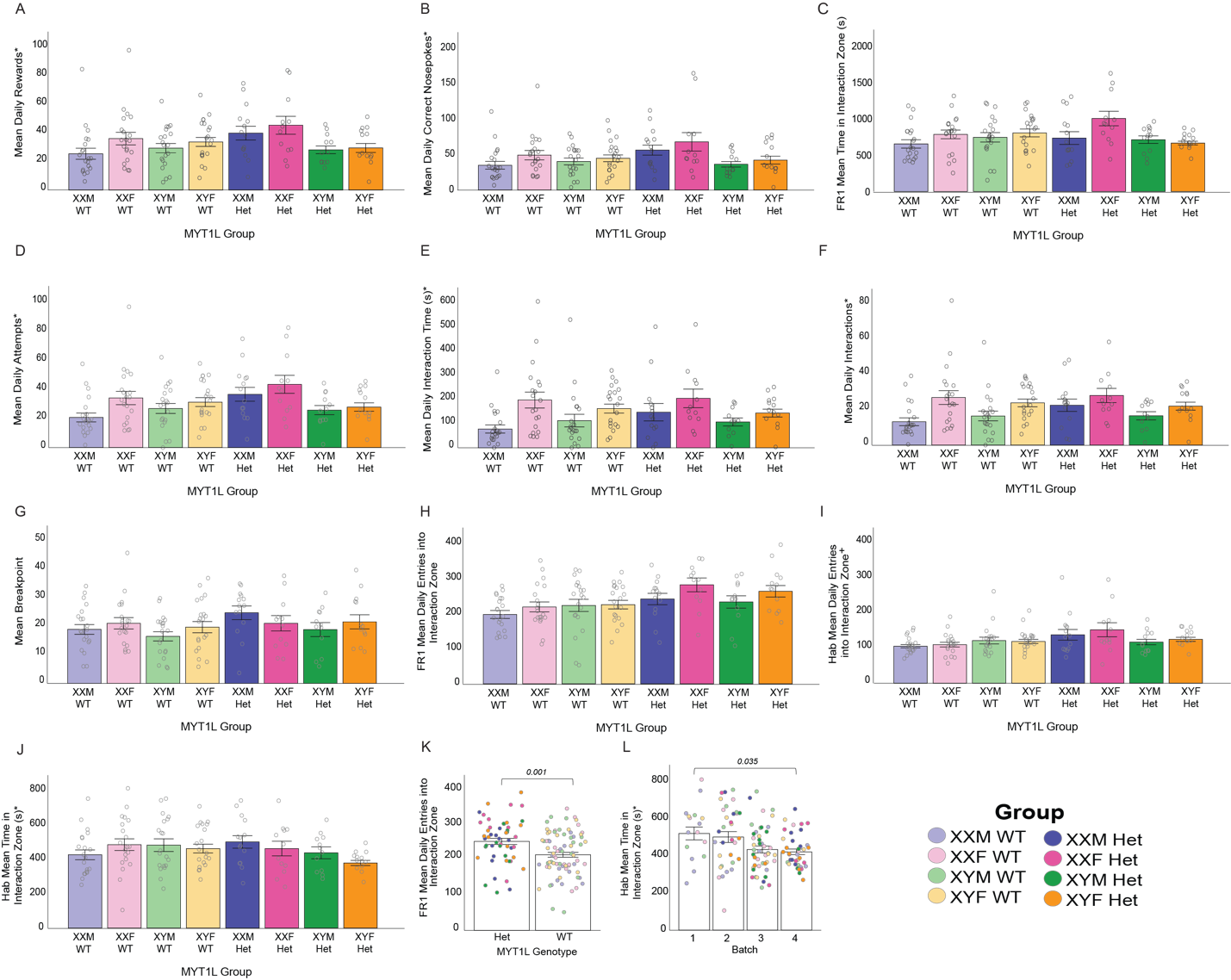


**Supplementary Figure 3: Gonadal and chromosomal sex independently act to alter social seeking and orienting in four core genotypes mice. A)** Mean Daily Rewards across all 8 groups. **B)** Mean Daily Correct Nosepokes across all 8 groups. **C)** Mean Time in the Interaction Zone across all 8 groups during FR1. **D)** Mean Daily Attempts across all 8 groups. **E)** Mean Daily Interaction Time across all groups. **F)** Mean Daily Interactions across all 8 groups. **G)** Mean breakpoint across all 8 groups, averaged from 3 separate PR trials. **H)** Mean Daily Entries into the Interaction Zone across all 8 groups during FR1. **I)** Mean Daily Entries into the Interaction Zone across all 8 groups during habituation. **J)** Mean Time in the Interaction Zone across all 8 groups during habituation. **K)** MYT1L Het test mice enter the interaction zone more often than MYT1L WT mice during FR1. **L)** Batch was the main driver of variation in Mean Time in the Interaction Zone during habituation, primarily driven by batch 4. For all panels, error bars indicate SEM. Asterisk (*) indicates variables that underwent square root transformation to normalize data distribution. Cross (^+^) indicates variables that underwent natural log transformation to normalize data distribution.

**
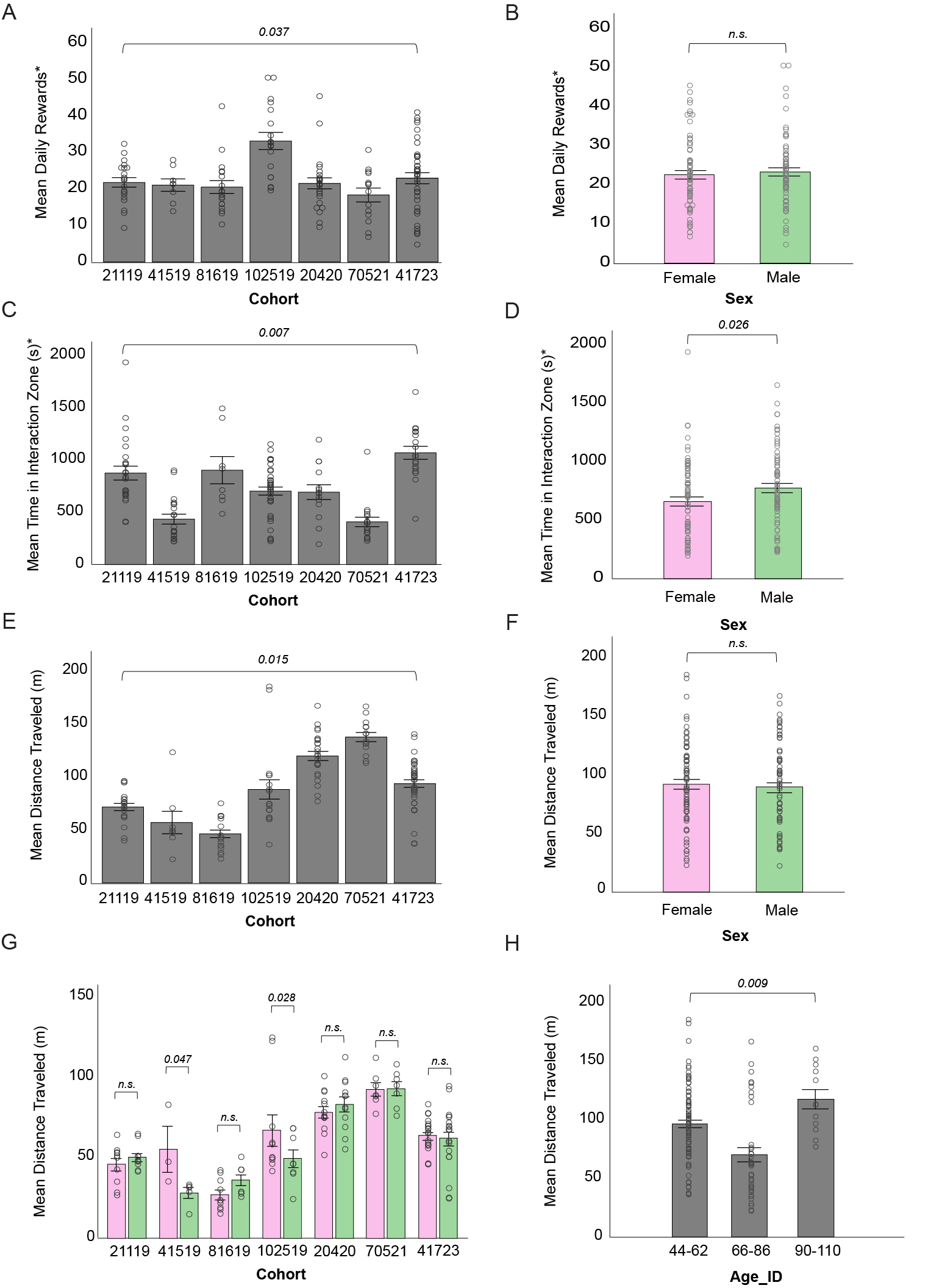
 Supplementary Figure 4: Mega-analysis shows variation in sex bias in social motivation is attributable to cohort effects. A)** Mean Daily Rewards for control groups across 7 social operant cohorts, with cohort effect shown**. B)** Mean Daily Rewards for 7 social operant cohorts split by sex shows no difference in social seeking. **C)** Mean Time in the Interaction Zone with cohort effect shown**. D)** Mean Time in the Interaction Zone Rewards for 7 social operant cohorts split by sex shows males spend more time in the interaction zone than females. **E)** Mean Distance Traveled with cohort effect shown**. F)** Mean Distance Traveled in the Interaction Zone Rewards for 7 social operant cohorts split sex shows no difference in activity. **G)** Mean Distance Traveled for each social operant cohort split by sex demonstrates two social operant cohorts (041519 and 102519) show significantly higher activity in females than males. **H)** Mean Distance Traveled was the only variable to have significant effects with age, namely driven by the 66-86 age group. Statistics indicate results from cohort subset of mega-analysis ANOVA and multiple comparisons (**Table S5)** and is not the same as reported sex bias from original published experiments **(Table S4)** which includes all experimental groups. For all panels, error bars indicate SEM. Asterisk (*) indicates variables that underwent square root transformation to normalize data distribution before analysis.

**Supplementary Table 1:**

| **Operant Interval** | **Social Motivation Component** | **Outcome** | **Definition** |
| --- | --- | --- | --- |
| FR1 | Social Seeking | Conditioning Criteria Achieved | Animal showed conditioning to reward stimulus for three consecutive days (40 correct nosepokes, 75% poke accuracy, and 75% successful rewards) |
| FR1 |  | Reward | Door opening in response to correct nosepoke in FR1 |
| FR1 |  | Correct Nosepokes | Nosepoke into active nose hole, including during a reward |
| PR3 |  | Breakpoint | Maximum nosepokes performed to elicit a reward in PR testing |
| Hab, FR1 | Social Orienting | Total Time in Interaction Zone | Duration of time in which the test mouse is in the interaction zone, regardless of the stimulus mouse or whether the door is open |
| Hab, FR1 |  | Entries into Interaction Zone | Number of entries the test mouse makes into the interaction zone, regardless of the stimulus mouse or whether the door is open |
| FR1 |  | Attempts | Number of rewards where the test mouse is at the interaction zone with the door open, regardless of stimulus mouse |
| FR1 |  | Interactions | Number of rewards where test and stimulus mice are both at the interaction zone with the door open |
| FR1 |  | Interaction Time | Duration of time the test and stimulus mouse are simultaneously at the open door during a reward |

Adapted from Maloney et al. 2023[9]. Interaction zone depicted in Figure 2.

Hab (Habituation); FR1 (Fixed Ratio 1); PR3 (Progressive Ratio 3

**Supplementary Table 2:**

| **Figure** | **Outcome** | **Method** | **Predictor** | **Output** | ***p* value** |
| --- | --- | --- | --- | --- | --- |
| 1 | OF Total Distance | ANOVA | Gonads | F(1,126) = 1.773 | 0.185 |
|  |  |  | Sex Chromosomes | F(1,126) = 11.100 | *0.001* |
|  |  |  | MYT1L | F(1,126) = 98.700 | *1.577E-17* |
|  | Soc Op Hab Mean Distance | ANOVA | Gonads | F(1,127) = 5.283 | *0.023* |
|  |  |  | Sex Chromosomes | F(1,127) = 0.001 | 0.981 |
|  |  |  | MYT1L | F(1,127) = 11.98 | *0.001* |
|  | Soc Op FR1 Mean Distance | ANOVA | Gonads | F(1,127) = 6.770 | *0.010* |
|  |  |  | Sex Chromosomes | F(1,127) = 2.143 | 0.146 |
|  |  |  | MYT1L | F(1,127) = 19.727 | *1.92E-5* |
| S1 | Heritability | Pearson’s Chi Square | MYT1L | X^2^(1) = 10.314 | *0.0013* |
|  |  | Pearson’s Chi Square | Tg(Sry)2Ei | X^2^(3) = 0.257 | 0.9678 |
|  | OF Perimeter Distance | ANOVA | Gonads | F(1,126) = 2.733 | 0.101 |
|  |  |  | Sex Chromosomes | F(1,126) = 8.725 | *0.004* |
|  |  |  | MYT1L | F(1,126) = 81.938 | *2.196E-15* |
|  | OF Center Distance | ANOVA | Gonads | F(1,127) = 0.023 | 0.879 |
|  |  |  | Sex Chromosomes | F(1,127) = 4.217 | *0.042* |
|  |  |  | MYT1L | F(1,127) = 36.314 | *1.695E-8* |
|  | OF Perimeter Entries | ANOVA | Gonads | F(1,127) = 0.275 | 0.601 |
|  |  |  | Sex Chromosomes | F(1,127) = 2.030 | 0.157 |
|  |  |  | MYT1L | F(1,127) = 24.531 | *2.282E-6* |
|  | OF Center Entries | ANOVA | Gonads | F(1,127) = 0.278 | 0.599 |
|  |  |  | Sex Chromosomes | F(1,127) = 1.993 | 0.160 |
|  |  |  | MYT1L | F(1,127) = 24.661 | *2.156E-6* |
|  | OF Time in Perimeter* | ANOVA | Gonads | F(1,127) = 0.083 | 0.773 |
|  |  |  | Sex Chromosomes | F(1,127) = 5.464 | *0.021* |
|  |  |  | MYT1L | F(1,127) = 1.638 | 0.203 |
|  | OF Time in Center* | ANOVA | Gonads | F(1,127) = 0.265 | 0.607 |
|  |  |  | Sex Chromosomes | F(1,127) = 5.092 | *0.026* |
|  |  |  | MYT1L | F(1,127) = 1.638 | 0.203 |
|  | OF Mean Time per Visit Center* | ANOVA *w/ simple main effects* | Gonads | F(1,126) = 0.040 | 0.842 |
|  |  |  | Sex Chromosomes | F(1,126) = 18.281 | *3.730E-5* |
|  |  |  | MYT1L | F(1,126) = 4.682 | *0.032* |
|  |  |  | Sex Chromosomes*Gonads | F(1,126) = 3.991 | *0.048* |
|  |  |  | *Testes, Sex Chromosomes* | F(1,126) = 19.814 | *1.857E-5* |
| 2 | Soc Op Mean Daily Total Rewards* | ANOVA *w/ simple main effects* | Gonads | F(1.126) = 5.281 | *0.023* |
|  |  |  | Sex Chromosomes | F(1,126) = 3.650 | 0.059 |
|  |  |  | MYT1L | F(1,126) = 3.144 | 0.079 |
| 3 |  |  | Sex Chromosomes*MYT1L | F(1,126) = 6.017 | *0.016* |
|  |  |  | *MYT1L Het, Sex Chromosomes* | F(1,126) = 7.780 | *0.006* |
|  |  |  | *XX, MYT1L genotype* | F(1,126) = 9.036 | *0.003* |
| 2 | Soc Op Mean Daily Interactions* | ANOVA | Gonads | F(1,127) = 19.130 | *3.7844E-7* |
|  |  |  | Sex Chromosomes | F(1,127) = 0.555 | 0.458 |
|  |  |  | MYT1L | F(1,127) = 1.802 | 0.182 |
| 2 | Soc Op Mean Daily Attempts* | ANOVA *w/ simple main effects* | Gonads | F(1,126) = 7.397 | *0.007* |
|  |  |  | Sex Chromosomes | F(1,126) = 2.608 | 0.109 |
|  |  |  | MYT1L | F(1,126) = 3.687 | 0.057 |
| 3 |  |  | Sex Chromosomes*MYT1L | F(1,126) = 5.940 | *0.016* |
|  |  |  | *MYT1L Het, SexChromosomes* | F(1,126) = 6.717 | *0.011* |
|  |  |  | *XX, MYT1L Genotype* | F(1,126) = 9.607 | *0.002* |
| 2 | Soc Op FR1 Mean Daily Total Time in Interaction Zone | ANOVA *w/ simple main effects* | Gonads | F(1,126) = 4.930 | *0.028* |
|  |  |  | Sex Chromosomes | F(1,126) = 1.648 | 0.202 |
|  |  |  | MYT1L | F(1,126) = 0.333 | 0.565 |
| 3 |  |  | Sex Chromosomes*MYT1L | F(1,126) = 5.987 | *0.016* |
|  |  |  | *MYT1L Het, Sex Chromosomes* | F(1,126) = 5.693 | *0.019* |
|  |  |  | *XX, MYT1L Genotype* | F(1,126) = 4.629 | *0.033* |
| S3 | Soc Op Hab Mean Daily Total Time in Interaction Zone* | ANOVA | Gonads | F(1,127) = 0.022 | 0.883 |
|  |  |  | Sex Chromosomes | F(1,127) = 0.125 | 0.725 |
|  |  |  | MYT1L | F(1,127) = 0.093 | 0.760 |
|  |  |  | Batch | F(3,127) = 2.957 | *0.035* |
| 2 | Soc Op Mean Daily Total Interaction Time* | ANOVA | Gonads | F(1,127) = 18.988 | *2.688E-5* |
|  |  |  | Sex Chromosomes | F(1,127) = 0.356 | 0.552 |
|  |  |  | MYT1L | F(1,127) = 1.130 | 0.290 |
| 3 | Soc Op Mean Daily Correct Nosepokes* | ANOVA *w/ simple main effects* | Gonads | F(1,126) = 4.167 | *0.043* |
|  |  |  | Sex Chromosomes | F(1,126) = 4.046 | *0.046* |
|  |  |  | MYT1L | F(1,126) = 3.482 | 0.064 |
|  |  |  | Sex Chromosomes*MYT1L | F(1,126) = 5.742 | *0.018* |
|  |  |  | *MYT1L Het, Sex Chromosomes* | F(1,126) = 7.948 | *0.006* |
|  |  |  | *XX, MYT1L Genotype* | F(1,126) = 9.192 | *0.003* |
| 2 | Soc Op Breakpoint | ANOVA | Gonads | F(1,127) = 0.935 | 0.335 |
|  |  |  | Sex Chromosomes | F(1,127) = 2.378 | 0.126 |
|  |  |  | MYT1L | F(1,127) = 3.065 | 0.082 |
| 3 | Soc Op Hab Mean Entries into Interaction Zone^+^ | ANOVA *w/ simple main effects* | Gonads | F(1,126) = 0.661 | 0.418 |
|  |  |  | Sex Chromosomes | F(1,126) = 0.042 | 0.838 |
|  |  |  | MYT1L | F(1,126) = 7.609 | *0.007* |
|  |  |  | Sex Chromosomes*MYT1L | F(1,126) = 5.149 | *0.025* |
|  |  |  | *XX, MYT1L genotype* | F(1,126) = 12.787 | *0.0005* |
| S3 | Soc Op FR1 Mean Entries into Interaction Zone | ANOVA | Gonads | F(1,127) = 3.568 | 0.061 |
|  |  |  | Sex Chromosomes | F(1,127) = 0.214 | 0.644 |
|  |  |  | MYT1L | F(1,127) = 11.868 | *0.001* |

Soc Op (Social Operant), OF (Open Field), Hab (Habituation), FR1 (Fixed Ratio 1)

(*) Indicates square root transformation to normalize distribution.

(^+^) Indicates natural log transformation to normalize distribution.

Statistics not shown are non-significant.

**Supplementary Table 3:**

Analysis on subset of consistent achievers (**Fig.S2B**)

| **Outcome** | **Method** | **Predictor** | **Output** | ***p* value** |
| --- | --- | --- | --- | --- |
| Soc Op Mean Daily Total Rewards* | ANOVA *w/ simple main effects* | Gonads | F(1,68) = 2.060 | 0.156 |
|  |  | Sex Chromosomes | F(1,68) = 9.540 | *0.003* |
|  |  | MYT1L | F(1,68) = 1.119 | 0.294 |
|  |  | Sex Chromosomes*MYT1L | F(1,68) = 4.323 | *0.041* |
|  |  | *MYT1L Het, Sex Chromosomes* | F(1,68) = 11.773 | *0.001* |
|  |  | *XX, MYT1L genotype* | F(1,68) = 4.686 | *0.034* |
| Soc Op Mean Daily Interactions* | ANOVA | Gonads | F(1,68) = 8.814 | *0.004* |
|  |  | Sex Chromosomes | F(1,68) = 2.308 | 0.133 |
|  |  | MYT1L | F(1,68) = 0.018 | 0.894 |
| Soc Op Mean Daily Attempts* | ANOVA *w/ simple main effects* | Gonads | F(1,68) = 2.030 | 0.159 |
|  |  | Sex Chromosomes | F(1,68) = 8.574 | *0.005* |
|  |  | MYT1L | F(1,68) = 0.381 | 0.539 |
|  |  | Sex Chromosomes*MYT1L | F(1,68) = 3.295 | 0.074 |
|  |  | *MYT1L Het, Sex Chromosomes* | F(1,68) = 9.915 | *0.002* |
|  |  | *XX, MYT1L Genotype* | F(1,68) = 2.814 | 0.098 |
| Soc Op Mean Daily Total Time in Interaction Zone | ANOVA *w/ simple main effects* | Gonads | F(1,68) = 1.160 | 0.285 |
|  |  | Sex Chromosomes | F(1,68) = 4.010 | *0.049* |
|  |  | MYT1L | F(1,68) = 0.621 | 0.434 |
|  |  | Sex Chromosomes*MYT1L | F(1,68) = 4.301 | *0.042* |
|  |  | *MYT1L Het,* Sex *Chromosomes* | F(1,68) = 7.336 | *0.009* |
|  |  | *XY, MYT1L Genotype* | F(1,68) = 4.281 | *0.042* |
| Soc Op Mean Daily Total Interaction Time* | ANOVA | Gonads | F(1,69) = 5.330 | *0.024* |
|  |  | Sex Chromosomes | F(1,69) = 3.663 | 0.060 |
|  |  | MYT1L | F(1,69) = 0.439 | 0.510 |
| Soc Op Mean Daily Correct Nosepokes* | ANOVA *w/ simple main effects* | Gonads | F(1,68) = 1.361 | 0.247 |
|  |  | Sex Chromosomes | F(1,68) = 9.566 | *0.003* |
|  |  | MYT1L | F(1,68) = 3.159 | 0.273 |
|  |  | Sex Chromosomes*MYT1L | F(1,68) = 3.159 | 0.080 |
|  |  | *MYT1L Het, Sex Chromosomes* | F(1,68) = 10.451 | *0.002* |
|  |  | *XX, MYT1L Genotype* | F(1,68) = 9.192 | 0.051 |
| Soc Op Mean Entries into Interaction Zone | ANOVA | Gonads | F(1,69) = 0.053 | 0.818 |
|  |  | Sex Chromosomes | F(1,69) = 0.471 | 0.495 |
|  |  | MYT1L | F(1,68) = 1.614 | 0.208 |

(*) Indicates square root transformation to normalize distribution; Soc Op (Social Operant)

Statistics not shown are non-significant.

**Supplementary Table 4:**

Summary of Cohorts Included in Social Operant Mega-Analysis

| **Cohort Number** | **Control Group** | **Age at start** | **Sample Size (M:F)** | **Sociability Bias in Original Analysis** |
| --- | --- | --- | --- | --- |
| 021119 | WT C57 | P56-82 | 10:10 | M > F |
| 081619 | WT C57 | P70-99 | 8:10 | M = F |
| 041519 | WT C57 | P58-75 | 5:3 | M = F |
| 102519 | WT C57 | P47-50 | 8:10 | M > F |
| 020420 | WT CD1 | P40-97 | 12:13 | M = F |
| 070521 | WT C57 | P70-99 | 7:7 | M > F |
| 041723 | XYM, XXF | P47-50 | 20:19 | M < F |

WT (Wildtype); M (Male); F (Female)

**Supplementary Table 5:**

Mega-Analysis on 7 published social operant cohorts **(Fig S3).**

| **Subset** | **Outcome** | **Method** | **Predictor** | **Output** | ***p* value** |
| --- | --- | --- | --- | --- | --- |
| Combined Controls | Mean Daily Rewards* | ANOVA *w/ cohort as random factor* | Sex | F(1,8.553) = 1.467 | 0.258 |
|  |  |  | Cohort | F(6,6) = 4.919 | *0.037* |
|  |  |  | Sex*Cohort | F(6,128) = 1.041 | 0.402 |
|  | Mean Total Time in Interaction Zone* | ANOVA *w/ cohort as random factor* | Sex | F(1,7.409) = 7.693 | *0.026* |
|  |  |  | Cohort | F(6,6) = 9.719 | *0.007* |
|  |  |  | Sex*Cohort | F(6,129) = 1.861 | 0.092 |
|  | Mean Distance | ANOVA *w/ cohort as random factor + simple main effects* | Sex | F(1,13.902) = 0.228 | 0.640 |
|  |  |  | Age | F(2,125) = 4.877 | *0.009* |
|  |  |  | *90-110 vs. 44-62* | 95% CI[905.129,4783.648] | *0.004* |
|  |  |  | Cohort | F(6,6) = 7.205 | *0.015* |
|  |  |  | Sex*Age | F(2,125) = 0.259 | 0.772 |
|  |  |  | Sex*Cohort | F(6,125) = 2.865 | *0.012* |
|  |  |  | *041519, Sex* | F(1,125) = 4.021 | *0.047* |
|  |  |  | *102519, Sex* | F(1,125) = 4.940 | *0.028* |
|  |  |  | *Female, Cohort* | F(6,125) = 16.877 | *3.336E-14* |
|  |  |  | *Male, Cohort* | F(6,125) = 10.788 | *1.195E-9* |

(*) Indicates square root transformation to normalize distribution.

Statistics not shown are non-significant.
